# Phytochemical Characterization of *Terminalia catappa* Linn. Extracts and Their antifungal Activities against *Candida* spp.

**DOI:** 10.3389/fmicb.2017.00595

**Published:** 2017-04-10

**Authors:** Analucia G. Terças, Andrea de Souza Monteiro, Eduardo B. Moffa, Julliana R. A. dos Santos, Eduardo M. de Sousa, Anna R. B. Pinto, Paola C. da Silva Costa, Antonio C. R. Borges, Luce M. B. Torres, Allan K. D. Barros Filho, Elizabeth S. Fernandes, Cristina de Andrade Monteiro

**Affiliations:** ^1^Departamento de Engenharia Elétrica, Programa de Doutorado em Biotecnologia da Rede Nordeste de Biotecnologia Universidade Federal do MaranhãoSão Luís, Brazil; ^2^Laboratório de Patogênese Bacteriana, Programa de Mestrado em Biologia Parasitária, Universidade CeumaSão Luís, Brazil; ^3^Laboratório de Micologia Médica, Programa de Mestrado em Odontologia, Universidade CeumaSão Luís, Brazil; ^4^Laboratório de Micologia Médica, Programa de Mestrado em Biologia Parasitária, Universidade CeumaSão Luís, Brazil; ^5^Departamento de Ciências Fisiológicas, Programa de Doutorado em Biotecnologia da Rede Nordeste de Biotecnologia, Universidade Federal do MaranhãoSão Luís, Brazil; ^6^Instituto de Botânica, Centro de Pesquisa em Ecologia e FisiologiaSão Paulo, Brasil; ^7^Programa de Pós-graduação, Universidade CeumaSão Luís, Brazil

**Keywords:** plant extracts, candidiasis, AIDS, antifungical agents, *Candida*, *Terminalia catappa*

## Abstract

*Terminalia catappa* Linn bark is used to treat dysentery by various populations in Southeast Asian countries, and its leaves have also been used in traditional medicine to treat hepatitis in India and the Philippines. Here, the antifungal actions of crude hydro-alcoholic extract (TcHE) and fractions from *T. catappa* leaves were assessed via the agar diffusion and microdilution tests on *Candida* reference strains and clinical isolates from patients with acquired immunodeficiency syndrome (AIDS). Additionally, the potential cytotoxic effects of TcHE were assessed on cultured human peripheral blood mononuclear cells (PBMC). *T. catappa* fractions and sub-fractions were analyzed by gas chromatography coupled to mass spectrometry with electron impact (GC/MS/EI), high-performance liquid chromatography coupled to mass spectrometry “electrospray” ionization in positive mode (HPLC/MS/MS/ESI^+^) and hydrogen nuclear magnetic resonance (^1^HNMR). TcHE and its fractions were able to inhibit the growth of all tested *Candida* strains with the *n*-butanol (FBuOH) fraction presenting the best antifungal activity. Testing of different FBuOH sub-fractions (SF) showed that SF10 was the most active against *Candida* spp. Fractioning of SF10 demonstrated that 5 out of its 15 sub-fractions were active against *Candida* spp., with SF10.5 presenting the highest activity. Chemical analysis of SF10 detected hydrolysable tannins (punicalin, punicalagin), gallic acid and flavonoid C-glycosides. Overall, the results showed that *T. catappa* L. leaf extract, fractions and sub-fractions were antifungal against *Candida* spp. and may be useful to treat diseases caused by this fungus.

## Introduction

The increase number of individuals infected with human immunodeficiency virus (HIV) has stimulated research regarding *Candida* spp., which are opportunistic pathogens responsible for one of the most common oral diseases affecting immunosuppressed individuals, the oropharyngeal candidiasis (OPC) (Hamza et al., 2008; Moura et al., 2010; Annapurna et al., 2012). Though Candida albicans is the most frequently isolated species as colonizer and pathogen of the oral mucosa, other Candida species, such as *Candida tropicalis, Candida krusei*, and *Candida glabrata* are recovered increasingly, mainly in HIV-infected individuals (Gugnani et al., 2003; Hamza et al., 2008; Patil et al., 2015). Candida yeasts cause oropharyngeal, esophageal, laryngeal, and invasive candidiasis (Patil et al., 2015).

Ten to 49% of HIV patients die due to invasive fungal infection (Favalessa et al., 2010). In addition, the constant use of antifungal drugs in the past few decades has increased the numbers of infection caused by resistant *Candida* strains. Thus, special attention has been given to research focused on medicinal plants to identify new molecules with antimicrobial potential (Fyhrquist et al., 2002; Jagessar and Alleyne, 2011; Packer et al., 2015).

Plants and their derived compounds have been used worldwide for the prevention and treatment of diseases (Triantafillidis et al., 2016). *Terminalia catappa* Linn. plant has been investigated in various pharmaceutical studies as it contains a variety of chemical components (Pandya et al., 2013; Yeh et al., 2014). *T. catappa* L. leaf extracts exhibit biological activities, including antioxidant (punicalagin, punicalin, terfluvina A and B, chebulic acid, benzoic acid, cumaric, and its derivatives) (Chen and Li, 2006; Chyau et al., 2006; Kinoshita et al., 2007), antidiabetic (β-carotene) (Anand et al., 2015), anticancer (punicalagin) (Naitik et al., 2012), antiviral (ellagic acid) (Tan et al., 1991), anti-inflammatory (triterpenic acids, especially ursolicacid and its derivatives) (Fan et al., 2004), antimicrobial (flavones and flavanols) (Kloucek et al., 2005; Nair and Chanda, 2008; Shinde et al., 2009), and hepato-protective activities (punicalagin, punicalin) (Kinoshita et al., 2007).

In India, a plaster of *T. catappa* leaves is used to treat scabies, leprosy wounds and other skin diseases (Nair and Chanda, 2008). Its traditional use includes the treatment of diarrhea and fever, especially in India, the Philippines and Malaysia (Kloucek et al., 2005; Shinde et al., 2009). Previous studies suggest that the most polar fractions obtained from *T. catappa* leaves are effective against bacteria (Shinde et al., 2009) and fungi (Jagessar and Alleyne, 2011), but little is known of *T. catappa* effects against *Candida* spp. Here, we investigated the antifungal properties of the hydroalcoholic extract obtained from *T. catappa* leaves (TcHE). We also assessed the antifungal actions of fractions and sub-fractions (SF) obtained from TcHE. Additionally, compounds of the most effective SF were identified.

## Materials and methods

### Plant materials and extraction

*T. catappa* Linn (Combretaceae) leaves were collected in São José de Ribamar, Maranhão, and identified by the Attic Herbarium Seabra of the Federal University of Maranhão (São Luís, Brazil) under the voucher specimen n°. 01062. The leaves were dried at room temperature for 1 week. The dried material was then triturated in a slicer to obtain a fine powder. Two hundred grams of powder were extracted twice with 600 ml of ethanol (70%, v/v) at room temperature for 2 days with an 8 h-period between extractions. The mixture was filtered through cellulose filter paper (Whatman No. 1, GE Healthcare UK, Amersham, UK) and concentrated on a rotary evaporator (Büchi Labortechnik AG, Flawil, Switzerland) under reduced pressure at 40°C.

The crude hydro-alcoholic extract (TcHE) was lyophilized, then (200 g) resuspended in 600 ml MeOH/H_2_O (Merck, Darmstadt, Germany) (80:20, v/v). The samples were sequentially subjected to liquid-liquid partition with hexane (Merck, Darmstadt, Germany), followed by ethyl acetate (Merck, Darmstadt, Germany), then *n*-butanol (Merck, Darmstadt, Germany), resulting in three fractions with different polarities: the hexane fraction (FHEX), which was the least polar fraction; the ethyl acetate fraction (FAcOEt) with intermediate polarity; and the *n*-butanol fraction (FBuOH), the most polar fraction. The resulting fractions were concentrated on a rotary evaporator (Büchi Labortechnik AG, Flawil, Switzerland), lyophilized (VirTis Lyophilizer Tray Dryer, New York, United States), weighed and stored in sterile glass containers at −20°C.

### Isolation and identification of *Candida* strains

*Candida* spp. samples were obtained from the buccal mucosa of 52 patients with acquired immunodeficiency syndrome (AIDS) attending the Hospital Presidente Vargas in São Luis, Brazil, after a written informed consent was obtained. Individuals (30 male and 22 female) were 19–61 years old. Samples were collected by rubbing sterile swabs on the oral mucosa, and were then inoculated into tubes containing 0.85% saline (ISOFAR, Paraná, Brazil). *Candida* spp. were isolated in CHROMagar (Difco, Detroit, MI, USA) and incubated at 37°C for 48 h. The biochemical identification of the yeast was performed in automated Vitek-2® system (Compact, bioMérieux, Marcy-L ‘Etoile, France), according to the manufacturer's recommendations. The study was reviewed and approved by the Ethics Committee of the Federal University of Maranhão (UFMA) under the protocol number 23115-006 540/2009-40.

### Monitoring of antifungal activity

#### Microdilution assay

The antifungal activities of TcHE, FHEX, FAcOEt, and FBuOH were evaluated in 20 clinical isolates (14 *Candida albicans*, 2 *Candida glabrata*, 2 *Candida tropicalis*, and 2 *Candida krusei*) and three reference strains (*C. albicans* ATCC 90028, *C. glabrata* ATCC 2001, and *C. krusei* ATCC 6258) of *Candida* spp. based on the proposal of the Clinical and Laboratory Standards Institute (CLSI) M27-A3 (CLSI, 2008). References strains were kindly donated by the São Paulo State University, Araraquara Dental School, São Paulo, Brazil. The antifungal effects of all FBuOH SFs (primary and secondary) were initially assessed against two clinical isolates of *C. albicans* (*C. albicans* 02 and *C. albicans* 04) and the reference strain *C. albicans* ATCC 90028 in the agar diffusion method using cavity plates (McGinnis, 1980). The effects of the most active SFs were also evaluated in the microdilution method, against *Candida* spp. reference strains and four clinical isolates (*C. albicans* 02, *C. glabrata* 01, *C. tropicalis* 01, *C. krusei* 02), one from each species of *Candida*.

In order to determine antifungal activities in the microdilution assay, the TcHE and its fractions were diluted in RPMI-1640 culture medium (Sigma-Aldrich, St. Louis, MO, USA) at concentrations of 0.18–48 mg/ml and were incubated in 96-well plates (at 37°C, for 48 h) with *Candida* spp. (2.5 × 10^3^ CFU/ml), as recommended by the CLSI M27-A3 protocol (CLSI, 2008). Fluconazole (0.25–64 μg/ml; Pfizer, Brazil) and amphotericin B (0.0625–16 μg/ml; Sigma-Aldrich, St. Louis, MO, USA) were used as positive controls. Vehicle (RPMI)-treated wells were used as negative controls. Following incubation, the absorbance was read at 540 nm in a microplate reader (BioMérieux Reader 250 Version 2.0.5, Genève, Suisse). All tests were performed in triplicate.

In a separate series of experiments, the most active SFs were tested at concentrations of 0.2–56 or 0.18–48 μg/ml, depending on their initial amount. In all experiments, the absorbance obtained from wells containing either TcHE, fractions or SFs plus medium and inoculum was discounted from those obtained from wells containing extract, fractions or SFs plus medium only. Then, the percentages of *Candida* spp. growth inhibition were determined for each strain using the formula outlined below:

$$\begin{array}{l} \%\text{ Inhibition}=\left(\text{Ac }-\text{ At}\right)/\text{Ac}\times 100 \end{array}$$

Where Ac = control absorbance and At = test absorbance.The MIC of each compound was defined as the lowest concentration at which no fungal growth was observed.

#### Agar diffusion assay

The antifungal effects of FBuOH SFs were evaluated in the agar diffusion method (Moody et al., 2004). Briefly, 20 ml of Sabouraud dextrose agar medium (SDA) (Difco Laboratories, Detroit, MI, USA) containing chloramphenicol were added to Petri dishes (100 × 200 mm in diameter) according to the manufacturer's recommendations. After solidification, the plates were incubated for 24 h at 37°C to assess contamination. Then, *Candida* spp. (100 μl of standardized inoculum solution with 10^3^ CFU/ml) was seeded on the plates with sterile swabs. Fifty-microliter extract volumes at different concentrations were added into perforations of approximately 5 mm in diameter in solid culture medium. Fluconazole (64 μg/ml) (Pfizer, SP, Brazil) and saline (0.9%) were used as positive and negative controls, respectively. All tests were performed in triplicate. The inhibition zones were measured in millimeters, and the data were analyzed by performing statistical tests.

### Human peripheral blood cell (PBMC) viability

PBMCs were collected from 20 healthy human volunteers (non-smoking donors who had not received any medication for the last 15 days prior to sampling, aged 18–35 years old) who provided written formal consent. This study was approved by the Ethics Committee in Research of Ceuma University (protocol number: 105/2014). Cells were obtained by the standard method of density-gradient centrifugation over Histopaque®-1119 according to the manufacturer's instructions. PBMCs were then suspended in a supplemented DMEM culture medium (Life Technologies, Brazil) containing 10% fetal bovine serum (Life Technologies, Brazil), streptomycin (100 μg/ml; Sigma–Aldrich, Brazil) and penicillin (100 U/ml; Sigma–Aldrich, Brazil). PBMCs were plated in 96-well plates (2 × 10^5^ cells/well in 200 μl) and incubated with either phosphate-buffered saline (PBS; negative control), or TcHE (0.001–10 mg/ml). Lipopolysaccharide (LPS)-treated wells were used as positive control. Cells were incubated at 37°C in a 5% CO_2_ atmosphere for 24 h. Then 100 μl of 3-(4,5-dimethylthiazol-2-yl)-2,5-diphenyl tetrazolium bromide (MTT, 2 mg/ml in PBS; Sigma-Aldrich, Brazil) were added to each well and the plates were incubated for 4 h at 37°C. Following incubation, the supernatants were removed and the formazan crystals formed were dissolved in 100 μl of 100% DMSO. Absorbances were read at 540 nm using a spectrophotometer. All experiments were performed three times in triplicate. Citotoxicity was then classified as follows: (i) 0 - not cytotoxic (inhibition below 25%); (ii) 1- slightly cytotoxic (inhibition between 25 and 50%); (iii) 2-moderately cytotoxic (inhibition between 50 and 75%) and (iv) 3-intensely cytotoxic (inhibition higher than 75%). Besides statistical analysis, the results were also evaluated in accordance with ISO standard 10993-5.

### Investigation of plant components

The crude hydro-alcoholic extract of *T. catappa* Linn, as well as FHEX, FAcOEt, and FBuOH, were subjected to phytochemical analysis based on the tests proposed by Matos (1998) to search for phenols, tannins, flavones, xanthones, flavonols, flavanoids, and triterpene steroids. This survey aimed to identify the classes of natural products (called secondary or functional plant metabolites) present in this extract.

### Fractionation of extracts using a sephadex® LH-20

After determining the antifungal activity of TcHE and its different fractions, the FBuOH (44.13 mg) fraction was sub-fractionated in a Sephadex® LH-20 (Sigma-Aldrich, St. Louis, MO, USA) column (5 × 100 cm) using methanol (Merck, Darmstadt, Germany) as the mobile phase. For this, the eluate was pumped at a flow rate of 500 ml/h for 4 h and then, 15 SFs were collected (25 ml/tube). The SFs exhibiting the greatest antifungal activities were identified, mixed and fractionated. The antifungal effects of these resulting SFs were also assessed against *Candida* spp. via the agar diffusion and microdilution methods.

### Chemical analysis

#### Gas chromatography coupled to mass spectrometry with electron impact ionization (GC/MS/EI)

TcHE, FHEX, FAcOEt, and FBuOH samples were derivatized with N,O-bis (trimethylsilyl) trifluoroacetamide with trimethylchlorosilane (BSTFA+TMCS, 99:1, v/v) (Sigma-Aldrich, St. Louis, MO, USA). The samples were analyzed using an Agilent 6890 gas chromatograph (Agilent Technologies, Inc., Santa Clara, CA) and mass spectrometry (Agilent 5973N MSD) with an HP-5 Agilent® (0.25 mm × 30 m × 0.25 μm) column under the following conditions: 230°C injection temperature, 250°C interface, 200°C ion source with a helium carrier gas flow of 1 ml/min. The column oven temperature increased as follows: 5 min of heating (70°C), gradient of 5°C/min up to 310°C.

#### High-performance liquid chromatography coupled with ultraviolet spectroscopy (HPLC/UV)

A total ion chromatogram (TIC) was obtained using an Agilent Varian ProStar HPLC system equipped with a ProStar 325 UV-Vis detector (ProStar 325, Varian, Palo Alto, USA) and a C18 column (Pursuit, Agilent Technologies, Inc., Santa Clara, CA, USA), which was protected with a guard column. For the mobile phase, pump A utilized Milli-Q water (Millipore-Merck, Darmstadt, Germany) acidified with 0.1% acetic acid; Pump C used HPLC-grade acetonitrile (Merck, Darmstadt, Germany) and pump B used methanol (HPLC grade, Merck, Darmstadt, Germany) to clean the column. The program used for elution was carried out from 0 to 15 min with gradient ranged from 95% A and 0.5% C to 72% A and 28% C. Then the gradient ranged from 72% A and 28% C to 32% A to 68% C in the interval between 16 and 55 min, reaching the concentration of 100% for C in the interval of 55–60 min. The detection of compounds of interest was performed at a wavelength (λ) of 254 nm, and the volume injected was 20 μl, with the mobile phase flow set at 0.6 ml/min.

#### Liquid chromatography coupled to electrospray ionization mass spectrometry in positive mode (HPLC-MS/MS/ES^+^)

This analysis was performed to determine the molecular masses of positive ions detected and to identify the components present in FBuOH and its sub-fractions. The analyses were conducted using a 10AD-VP chromatographic system coupled with a Shimadzu SPD-M10AVP DAD detector (Shimadzu, Kyoto, Japan) and an Esquire 3,000 Plus-Ion Trap mass spectrometer (Bruker Daltonics, GmbH, Bremen, Germany) equipped with a 4,000 V capillary, a nebulizer set at 27 psi, a drying gas flux of 7 l/min, and a temperature of 320°C, in positive ion mode.

#### Nuclear magnetic resonance of hydrogen (^1^HNMR)

To identify the compounds present in FBuOH sub-fraction 10 (SF10-FbuOH), this sub-fraction was subjected to analysis via ^1^H nuclear magnetic resonance using a 300-MHz Varian Mercury Plus spectrometer (Varian Medical Systems, Inc., CA, USA) (7.05 T) in CD_3_OD. Chemical shifts (δ) were reported in ppm using tetramethylsilane (TMS) as an internal reference standard, and coupling constants (J) were given in Hz. The ATB (Automation Triple Resonance Broadband) probe (5 mm inner diameter) was used at room temperature and pulse 45 to detect ^1^H.

### Statistical analysis

Each experiment was performed in triplicate and results were expressed as mean ± standard deviation (SD). To evaluate the effects of the various fractions and sub-fractions on fungal growth, we initially applied the Shapiro-Wilk test for normality. Once the results were confirmed to be normally distributed, the data were analyzed by ANOVA followed by Tukey's test. Data obtained from PBMC cell viability test were analyzed by ANOVA followed by Dunnet's test. The significance level was set at 5% for all tests.

## Results

### Distribution of *Candida* species from mucosal tissues

This study included 52 patients diagnosed with AIDS. *Candida* cells were found in the oral cavities of 83% (43) patients, with some of them (18.6%) colonized with multiple *Candida* spp. As a result, 51 yeast strains were isolated. Of those, 56% were *C. albicans* (29/51), 12% were *C. tropicalis* (6/51), 12% were *C. krusei* (6/51), 8% were *C. glabrata* (4/51), 4% were *C. famata* (2/51), 4% were *C. parapsilosis* (2/51), and 4% were *C. guilliermondii* (2/51). The four most prevalent isolates of *Candida* spp. were investigated in the antifungal activity assays.

### Plant material: yield and phytochemical analysis

Yields obtained for TcHE, FHEX, FAcOEt, and FBuOH were depicted in Table 1 with FBuOH presenting the lowest one. *T. catappa* TcHE, FHEX, FAcOEt, and FBuOH were subjected to phytochemical screening according to the method described by Matos (1998). The analysis revealed the presence of phenols, tannins, flavones, flavonols, flavonoids, and triterpenes sterols (Table 2).

**Table 1:** **The dry weights and yields of the hydro-alcoholic extract and its fractions**.

**Weight of dry leaves**	**TcHE**	**FHEX**	**FAcOEt**	**FBuOH**
1 kg (570 ml)	46.17 g	2.123 g	2.317 g	1.067 g
Yield	5.77%	0.26%	0.28%	0.13%

*TcHE, crude hydro-alcoholic extract of T. catappa L.; FHEX, hexane fraction; FAcOEt, ethyl acetate fraction; FBuOH, N-butanol fraction*.

**Table 2:** **Phytochemical analysis of the hydro-alcoholic extract and its fractions**.

**Compounds class**	***Terminalia catappa* extracts**			
	**TcHE**	**FHEX**	**FAcOEt**	**FBuOH**
Phenols	+	+	+	+
Tannins	+	−	+	+
Flavones, xanthones, flavonols	+	+	−	+
Flavonoids	+	−	−	+
Triterpenes steroids	+	+	−	−

*TcHE, crude hydro-alcoholic extract of T. catappa L.; FHEX, hexane fraction; FAcOEt, ethyl acetate fraction; FBuOH, N-butanol fraction; (+), presence of substance; (−), absent of substance*.

### Antifungal activity

Antifungal assays were performed against different clinical and reference strains of *Candida*. Both the TcHE and its fractions were antifungal against all the tested strains of *Candida* when assessed in the microdilution method (Table 3). However, their antifungal potencies varied between strains. MIC values ranged from 1.5 to 6 mg/ml for FHEX, 0.75 to 3 mg/ml for FBuOH, 0.75 to 6 mg/ml for FAcOEt and 0.75 to 12 mg/ml for TcHE (Table 3). The MIC_90_ (minimum concentration able to inhibit 90% of strains) for TcHE, FHEX and FAcOEt was 3 mg/ml, but 1.5 mg/ml for FBuOH. FBuOH presented the lowest MIC values (Table 3).

**Table 3:** **Minimum inhibitory concentrations of amphotericin B, fluconazole, FHEX, FBuOH, FAcOEt, TcHE against *Candida* spp**.

**Strains**	**Amphotericin B (μg/ml)**	**Fluconazole (μg/ml)**	**FHEX (mg/ml)**	**FBuOH (mg/ml)**	**FAcOEt (mg/ml)**	**TcHE (mg/ml)**
*C. albicans* 01	2	16	6	1.5	3	3
*C. albicans* 02	1	16	3	3	3	12
*C. albicans* 03	2	8	3	1.5	1.5	1.5
*C. albicans* 04	2	4	3	1.5	1.5	1.5
*C. albicans* 05	2	8	3	1.5	3	1.5
*C. albicans* 06	2	8	1.5	1.5	1.5	1.5
*C. albicans* 07	2	4	1.5	1.5	1.5	3
*C. albicans* 08	2	8	3	0.75	1.5	3
*C. albicans* 09	1	4	3	1.5	6	12
*C. albicans* 10	1	4	3	3	3	3
*C. albicans* 11	2	16	6	3	6	6
*C. albicans* 12	4	8	6	3	3	3
*C. albicans* 13	2	16	1.5	0.75	1.5	3
*C. albicans* 14	1	16	6	1.5	3	3
*C. glabrata* 01	2	16	1.5	1.5	1.5	3
*C. glabrata* 02	2	16	1.5	1.5	1.5	1.5
*C. tropicalis* 01	2	32	3	1.5	3	3
*C. tropicalis* 02	2	32	3	1.5	3	3
*C. krusei* 01	4	>64	1.5	1.5	1.5	2
*C. krusei* 02	4	32	1.5	1.5	1.5	3
*C. albicans* ATCC 90028	2	16	6	1.5	1.5	1.5
*C. glabrata* ATCC 2001	2	16	6	1.5	0.75	0.75
*C. krusei* ATCC 6258	0.5	16	3	0.75	0.75	0.75
MIC_50_^*^	2	16	3	1.5	1.5	1.5
MIC_90_^**^	2	16	3	1.5	3	3
MIC range	1–4	4–> 64	1.5–6	0.75–3	0.75–6	0.75–12

* *MIC_50_, MIC value that inhibited 50% of the strains*.

** *MIC_90_, MIC value that inhibited 90% of the strains*.

**Figure 1:**
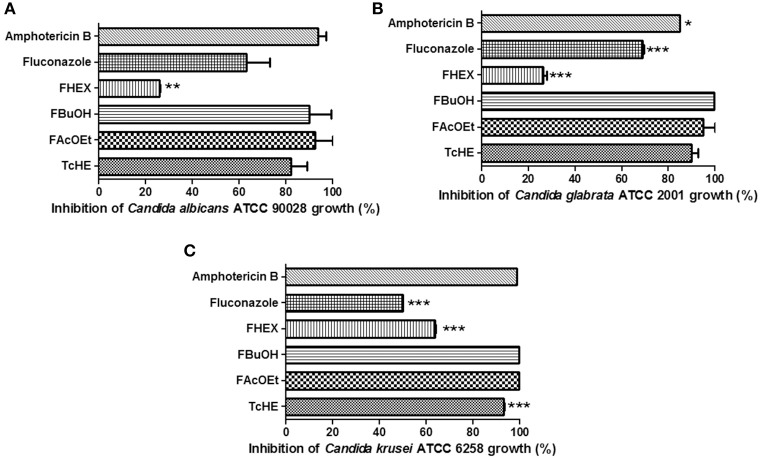
**Inhibitory effect of fluconazole and amphotericin B (clinically used antifungal agents), TcHE, FAcOEt, FBuOH, and FHEX on *Candida* reference strains growth. (A)** *Candida albicans* ATCC 90028; **(B)** *Candida glabrata* ATCC 2001; **(C)** *Candida krusei* ATCC 6258. The concentrations used were: 1.5, 8, and 2 μg/ml for TcHE and its fractions, fluconazole and amphotericin B, respectively. Inhibitory action of each substance was compared to that of FBuOH. ^*^p < 0.05, ^**^p < 0.01, and ^***^p < 0.0001 were considered to be significant.

Amphotericin B, fluconazole, FHEX, FAcOEt, and TcHE were compared to FBuOH in relation to the capacity of *Candida* growth inhibition. Growth inhibition assays showed that TcHE, FBuOH, and FAcOEt (1.5 mg/ml) presented the best antifungal activities against all the tested reference strains of *Candida* spp with percentage of inhibitions as high as 100% (Figures 1A–C). Significant differences were observed across fractions with FHEX being the least active (*p* < 0.01 or *p* < 0.0001 depending on species, Figures 1A–C). Of note, TcHE, FBuOH, and FAcOEt were more effective than fluconazole (*p* < 0.0001) and amphotericin B (*p* < 0.05) (Figures 1A–C). *C. krusei* ATCC 6258 was sensitive dose dependent (SDD) to fluconazole (Figure 1C; MIC value of 16 μg/ml).

When tested against *C. albicans* ATCC 90028, MIC values were of 6 mg/ml for FHEX, and 1.5 mg/ml for FBuOH, FAcOEt, and TcHE. Also, MIC values were of 6 mg/ml for FHEX, 1.5 mg/ml for FBuOH, and 0.75 mg/ml for FAcOEt and TcHE against *C. glabrata* ATCC 2001. FHEX (3 mg/ml), FBuOH, FAcOEt, and TcHE (0.75 mg/ml) were all able to inhibit *C. krusei* ATCC 6258 growth (Table 3).

**Figure 2:**
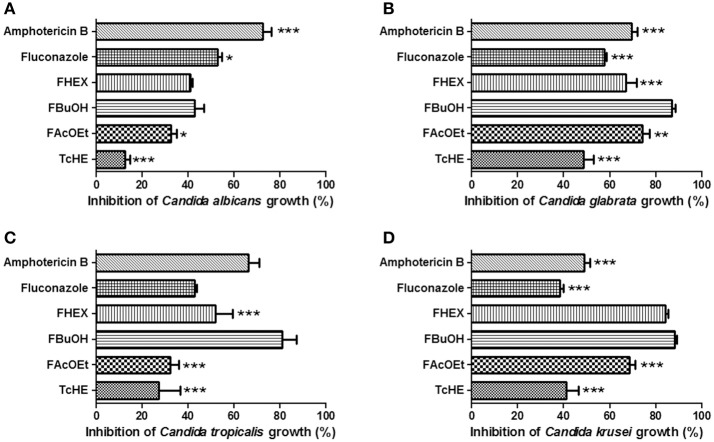
**Inhibitory effect of fluconazole and amphotericin B (clinically used antifungal agents), TcHE, FAcOEt, FBuOH, and FHEX on *Candida* clinical samples growth. (A)** *Candida albicans*; **(B)** *Candida glabrata*; **(C)** *Candida tropicalis*; **(D)** *Candida krusei*. The concentrations used were: 1.5, 8, and 2 μg/ml for TcHE and its fractions, fluconazole and amphotericin B, respectively. Inhibitory action of each substance was compared to that of FBuOH. ^*^p < 0.05, ^**^p < 0.01, and ^***^p < 0.0001 were considered to be significant.

Figures 2A–D represented some of the best results of antifungal potential of TcHE and its fractions against four clinical isolates, one from each species, at 1.5 mg/ml. FBuOH showed better antifungal activity than TcHE (*p* < 0.0001) and FAcOEt (*p* < 0.05) against *C. albicans* clinical strain (Figure 2A). Amphotericin B (*p* < 0.0001) and fluconazole (*p* < 0.05) presented higher activity in comparison to that observed for FBuOH (Figure 2A). Similarly, FBuOH presented the best activity on *C. glabrata* and *C. tropicalis* clinical isolates (*p* < 0.0001, Figures 2B–C). These effects were even more pronounced than those observed for either fluconazole or amphotericin B (*p* < 0.0001, Figures 1B,C). When evaluated against *C. krusei* clinical isolates, FBuOH and FHEX were the most effective, followed by FAcOEt (*p* < 0.0001, Figure 2D). *C. krusei* and *C. tropicalis* clinical samples were SDD to fluconazole (Figures 2C–D), with MIC values of 16 μg/ml (Table 3).

**Figure 3:**
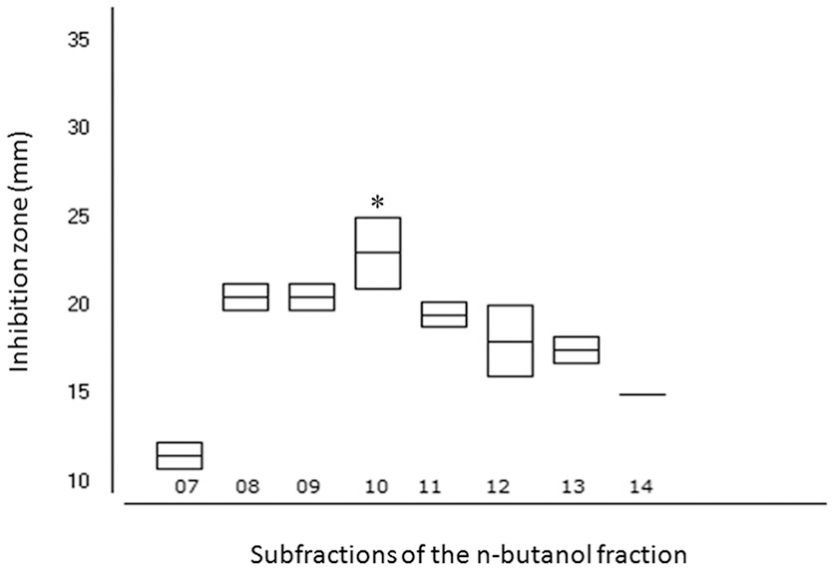
**Boxplots of the mean values and standard deviations of the sizes of the inhibition zones caused by activity of specific FBuOH sub-fractions (SF 7–14)**. ANOVA and Tukey's test (*p* < 0.001) showed that the SF10-FBuOH had a significant greater inhibition zone size than the other sub-fractions tested (^*^p < 0.05).

As FBuOH was found to exhibit the best antifungal activity, we next assessed the antifungal properties of its sub-fractions (SF 1–15), by using the agar diffusion test. Figure 3 illustrates the test results for a clinical isolate. Of those sub-fractions, only SF 7–14 were antifungal (112 μg/ml) (Figure 3). SF 10 (SF10-FBuOH) presented the largest inhibition zone (*p* < 0.001, Figure 3). We next assessed the antifungal actions of SF10-FBuOH (0.2–56 μg/ml) in the microdilution test against four clinical isolates and reference strains.

SF10-FBuOH MIC values ranged from 7 to 56 μg/ml, with the lowest value being registered against the *C. albicans* isolate. Both the MIC_50_ (minimum concentration able to inhibit 50% of strains) and the MIC_90_ (minimum concentration able to inhibit 90% of strains) were 28 μg/ml (**Table 5**).

SF10-FBuOH was then fractionated and the resultant sub-fractions (SF 10.1–10.10) were evaluated in the agar diffusion method against *C. albicans* ATCC 90028 and two *C. albicans* clinical isolates. Only SF 10.5–10.10 were antifungal, with SF 10.5 being the most active sub-fraction (*p* < 0.05). Table 4 depicted the results when SF 10.5–SF 10.10 were tested at 96 μg/ml. This antifungal action was further confirmed in the microdilution test. SF 10.5 MIC values ranged from 6 to 48 μg/ml with the lowest value being registered against the *C. tropicalis* isolate. MIC_50_ was of 24 μg/ml and MIC_90_ was 48 μg/ml (Table 5).

**Table 4:** **Antifungal potential of sub-fractions SF 10.5, SF 10.6, SF 10.8, SF 10.9, and SF 10.10, derived from sub-fraction SF10-FBuOH, evaluated by the antifungal agar diffusion test against *Candida albicans* ATCC 90028 and two clinical specimen of *Candida albicans***.

***C. albicans* strains**	**SF10 sub-fractions**					
	**SF 10.5**	**SF 10.6**	**SF 10.8**	**SF 10.9**	**SF 10.10**	**FLZ**
ATCC 90028	15 ± 0.81	12.33 ± 1.24	11.33 ± 1.24	9.67 ± 0.47	8.67 ± 1.24	6.33 ± 0.44
*Ca* 02	15 ± 1.0	14 ± 2.64	14.66 ± 1.52	10.66 ± 1.15	9.97 ± 0.46	5.33 ± 1.25
*Ca* 04	15.33 ± 1.25	11.67 ± 1.24	10.66 ± 1.69	9.33 ± 1.24	9.33 ± 0.47	10.43 ± 0.62

*Inhibition zones size is expressed in mm; FLZ, fluconazole (64 μg/ml)*.

*The antifungal activity was obtained from three independent experiments done in triplicate at a concentration of 96 μg/ml*.

*The ANOVA test was applied where each value represents the mean ± the standard deviation (SD) of the inhibition zones size (mm)*.

**Table 5:** **Minimum inhibitory concentration (μg/mL) of amphotericin B, fluconazole, SF10-FBuOH and SF- 10.5 against *Candida* spp**.

**Strains**	**Amphotericin B**	**Fluconazole**	**SF10-FBuOH**	**SF-10.5**
*C. albicans* 02	8	16	7	24
*C. glabrata* 01	8	16	28	24
*C. tropicalis* 01	8	32	28	6
*C. krusei* 02	8	>64	28	48
*C. albicans* ATCC 90028	8	32	28	24
*C. glabrata* ATCC 2001	8	64	14	24
*C. krusei* ATCC 6258	4	16	14	48
MIC_50_^*^	8	32	28	24
MIC_90_^**^	8	64	28	48
MIC range	4–8	16–> 64	7–28	6–48

* *MIC_50_, MIC value that inhibited 50% of the strains*.

** *MIC_90_, MIC value that inhibited 90% of the strains*.

### PBMC viability

**Figure 4:**
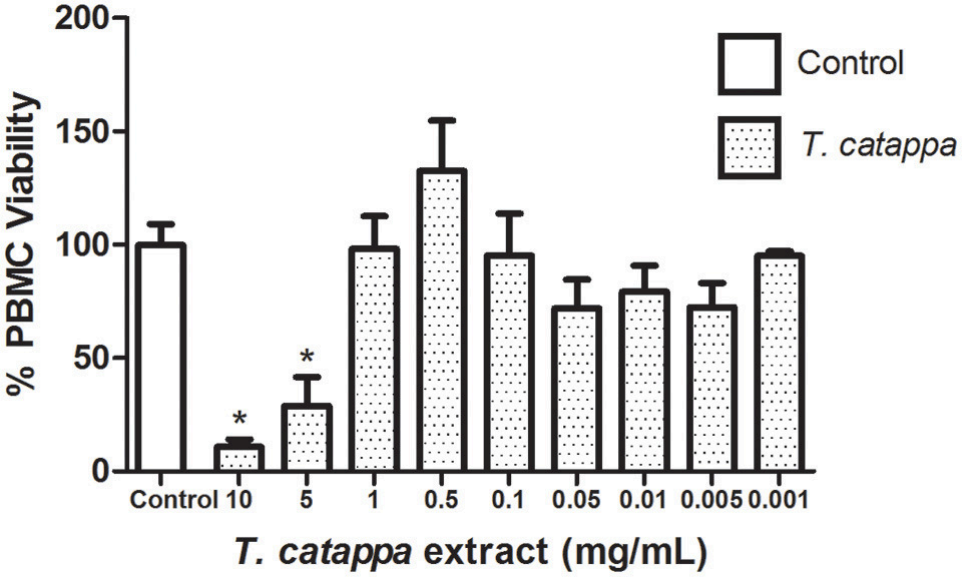
**PBMC cell viability after exposure to different concentrations of TcHE**. Symbol (^*^) represents statistical differences between the control and experimental groups (ANOVA one way followed by Dunnet test, *p* < 0.05).

The cytotoxic effects of TcHE were evaluated on PBMC. Overall, TcHE was not cytotoxic, only reducing cell viability at very high concentrations (5–10 mg/ml) in comparison with vehicle-treated controls (*p* < 0.05, Figure 4).

### Chemical analysis and identification of the components of FBuOH and SF10-FBuOH

Total ion chromatography (TIC) of SF10-FBuOH revealed a chromatographic peak with a retention time of 30 min corresponding to gallic acid. The probability of this compound being gallic acid was 90% according to the NIST/EPA/NIH Mass Spectral Library (National Institute of Standards and Technology, Gaithersburg, MD).

**Figure 5:**
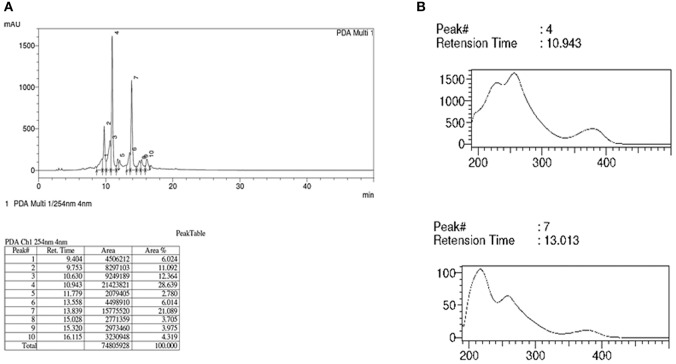
**Analysis of LC/DAD and HPLC/DAD data for SF10-FBuOH. (A)** The chromatographic profile and a table containing the RTs and areas (%) of peaks detected at 254 nm. HPLC detected 10 peaks in FBuOH, with RTs ranging from 9.404 to 16.115 min. The major peaks were peaks 4 (RT = 10.943 min) and 7 (RT = 13.013 min). **(B)** The ultraviolet absorption spectra of the major peak was at 4:07.

**Figure 6:**
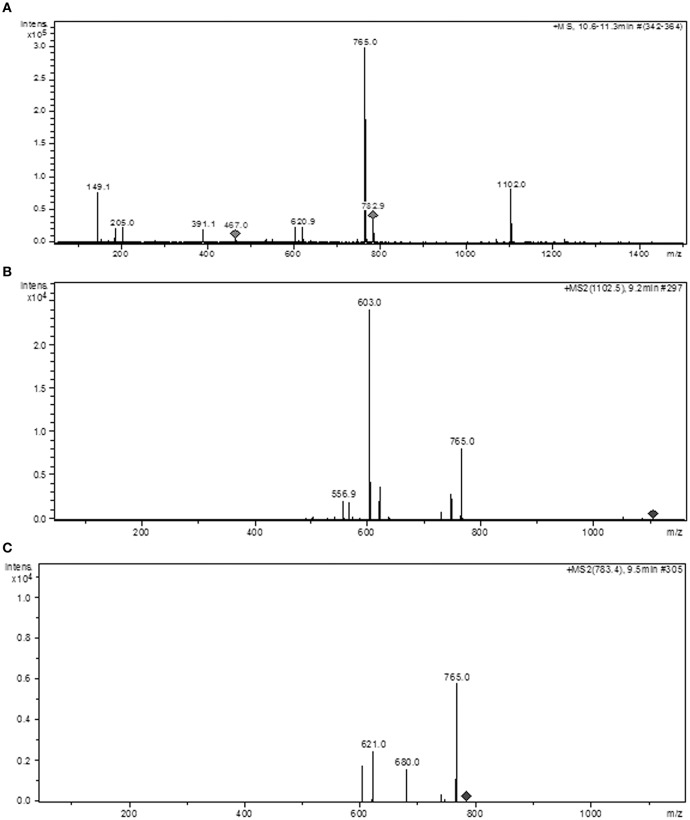
**An analysis of the LC/MS/MS/IES data for SF10-FBuOH. (A)** MS2 with ions detected by TIC. **(B)** The MS2 peak with RT = 9.2 min (punicalagin). **(C)** The EM2 peak with RT = 9.5 min (punicalin).

Analysis of FBuOH using the HPLC-DAD/MS/MS/ESI^+^ system revealed 10 chromatographic peaks with two major peaks: peak number 4, with a retention time (Rt) of 10.943 min (28.639%) and peak 7 with a Rt of 13.839 min (21.089%) (Figure 5). These peaks were accompanied by their respective absorption spectra in the ultraviolet region (Figure 6). The maximum wavelengths (maxλ) of the absorption bands for all peaks were characteristic of the galagil chromophore, at 218, 260, and 379 nm and demonstrated varying intensities for the absorption band at 218 nm (Figure 6). Thus, FBuOH of *T. catappa* is rich in compounds found in the ellagitannins (hydrolyzable tannins) class.

**Figure 7:**
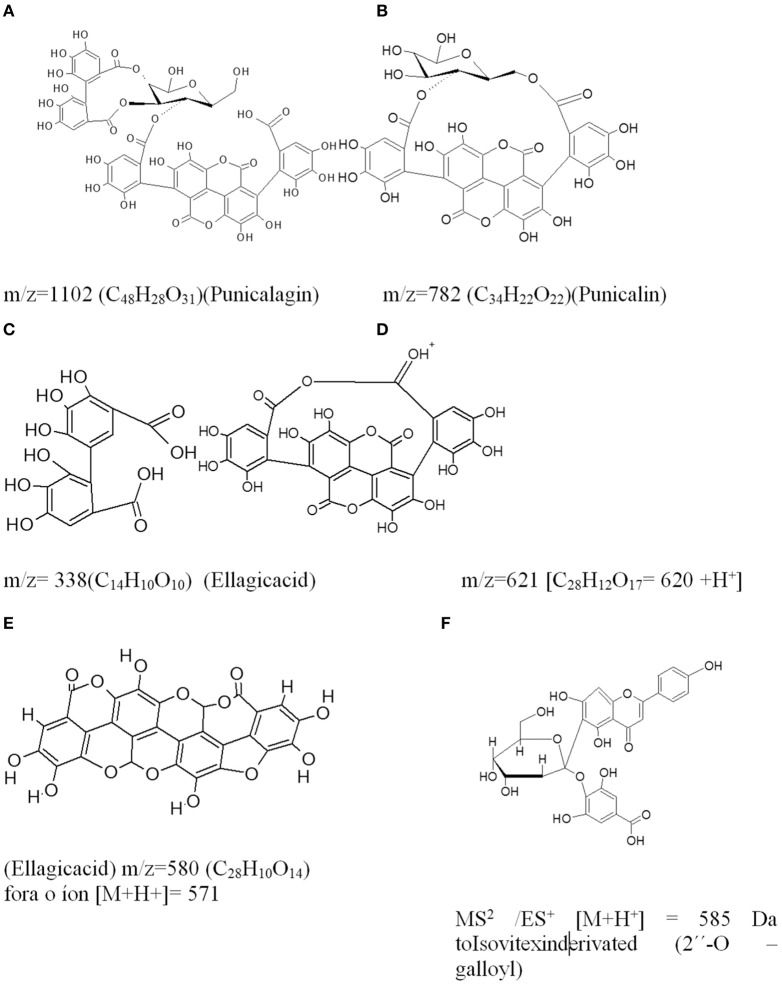
**Structures proposed by the results of the MS/MS/ES^+^ analysis for ions detected at RTs ranging from 9.2 to 11.5 min in SF10-FBuOH**. **(A)** The pseudomolecular ion [M+H^+^] = 1,103 (RT = 9.2 min) and molecular mass of M = 1,102 for the molecular formula C_48_H_28_O_31_, which was identified as an ellagitannin acid derivative of punicalagin. **(B)** The pseudomolecular ion [M+H^+^] = 783 (RT = 9.3 min) with a molecular mass of M = 782 for molecular formula C_34_H_22_O_22_, identified as punicalin. **(C)** The pseudomolecular ion [M+H^+^] = 338 M for the formula C_14_H_10_O_10_, which was identified as ellagic acid dihydrate. **(D,E)** Ellagic acid derivative. **(F)** Isovitexin derivative (2″-O –galloyl).

The MS1/MS2/ES^+^ system was used in FBuOH and SF10-FBuOH, which allowed us to determine the molecular weights of the positive ions detected and to identify the components in sub-fractions rich in ellagitannins and other classes of compounds, such as C-flavonoid glycosides (Figure 7). In the chromatogram for FBuOH, the ion [M+H^+^] detected at the MS RT of 9.2 min showed a mass to charge (*m*/*z*) ratio of [M+H^+^] = 1.103, which was compatible with the molecular formula C_48_H_30_O_31_ for which M = 1,102 Da, which was identified as an ellagitannin acid derived from punicalagin. The ion [M+H^+^] detected at the MS1 RT of 9.5 min had an *m*/*z* ratio of [M+H^+^] = 783, detected for fragments with *m*/*z* = 765 (100%), *m*/*z* = 680 and *m*/*z* = 621. This mass was compatible with the molecular formula C_34_H_22_O_22_, for which M = 782 Da, and was identified as an ellagitannin acid derivative of punicalin.

**Figure 8:**
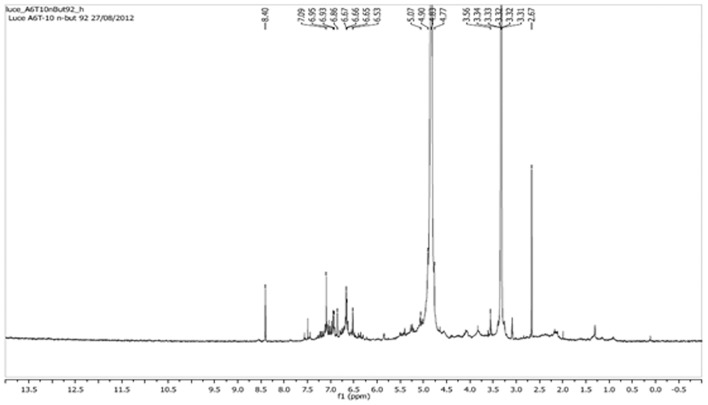
**The nuclear magnetic resonance spectrum of hydrogen (^1^H) detected using a 300-MHz Varian instrument in CD3 OD**. The chemical shift signals (δ or dpi) are shown for SF10-FBuOH.

The structural formulas proposed for the compounds in FBuOH were shown in Figure 8 based on LC/MS/ES^+^ analysis of the ions detected with retention times in the range of 9.2–11.5 min in FBuOH.

The ^1^HNMR spectra demonstrated the chemical shift of signals (δ, or ppm), with data for SF10-FBuOH revealing two additional intense signals at δ = 3.3 and 4.8, which were derived from the CH_3_OH and H_2_O non-deuterated solvents. The signal in the δ = 6.4–6.82 region corresponded to hydrogens of the aromatic ring, and the signal in the δ = 3.98–4.2 region referred to the methinic hydrogens of a sugar moiety, while δ = 2.15 in the form of multiple signals was assigned to the sugar methylene group (CH_2_) (Figure 8).

The expansion of the ^1^HNMR spectra of the δ 8.4–3.8 region revealed signals of chemical shift at δ = 8.5 attributed to one acid hydroxyl (OH), confirming the presence of free carboxyl groups for the structure with M = 1,102 Da. Signals with different coupling constants were also identified in the δ 5.5–4.9 region which could be attributed to the anomeric hydrogens of the α β structures derived from punicalin and punicalagin. Therefore, chromatographic studies of FBuOH and its sub-fractions revealed a compound formed by hydrolyzable tannins (punicalin and punicalagin), gallic acid and C-flavonoid glycosides.

## Discussion

This study confirmed the high frequency of oral candidiasis in hospitalized patients with AIDS (83%), as reported in other studies, demonstrating the development of one or more fungal infections in 80–95% of patients during the course of their illness (Bouza and Muñoz, 2008; Junqueira et al., 2012). *C. albicans* was the most prevalent species (56%), while the prevalence of all “non-*albicans*” *Candida* spp. was 44%, which is in agreement with other studies (Favalessa et al., 2010; de Paula et al., 2015).

In general, patients with HIV/AIDS manifest approximately five episodes of oral candidiasis per year (Favalessa et al., 2010). Frequent episodes of fungal infection in immunosuppressed patients and the use of antifungal drugs have selected for fungal strains resistant to antifungal drugs. In this study, fluconazole inhibited less than 50% of all *Candida* strains tested, confirming the development of fungal resistance in hospitalized patients with AIDS.

The increased incidence of antimicrobial-resistant *Candida* isolates has highlighted the need for novel and more potent therapies against this microorganism. *T. catappa* L. is a plant species that contains a wide variety of chemical components (Lin et al., 2001; Kinoshita et al., 2007; Naitik et al., 2012) and is widely used in the traditional medicine in India, Malaysia, and Indonesia. However, studies addressing the antifungal properties of *T. catappa* are scarce and little is known on the anti-*Candida* activities of hydro-alcoholic extracts obtained from *T. catappa* leaves. Recently, the crude ethanolic or aqueous extract from the leaves of *T. catappa* L. was suggested to be more effective against bacteria than *Candida* spp. (Jagessar and Alleyne, 2011). This poor effect was attributed to the lower concentration of substances active against filamentous fungi and *C. albicans* (Jagessar and Alleyne, 2011; Mandloi et al., 2013) in these preparations. Herein, we present novel and important evidence on that the TcHE is antifungal against *Candida* spp., and that this effect is due to the presence of antimicrobial compounds in the FBuOH fraction. Also, TcHE had not cytotoxic actions on PBMC.

The results obtained demonstrate that FBuOH and FAcOEt are more effective than TcHE and FHEX in reducing *Candida* spp. growth (>90%). Of note, FBuOH actions were greater than those observed for FAcOEt, as it presented the lowest MIC values. Accordingly, fractionation of FBuOH showed its sub-fractions SF7–14 present potent antifungal activities, specially, SF10-FBuOH; suggesting the compounds recovered in this sub-fraction exhibit superior antifungal activity. The antifungal activity of SF10-FBuOH was further confirmed by isolation of SF10.5, a sub-fraction of SF10-FBuOH which presented potent antimicrobial actions against *Candida* spp.

The chemical analysis of SF10-FBuOH detected the presence of hydrolysable tannins (punicalin and punicaligin), gallic acid and C-flavonoid glycosides. It was previously demonstrated that hydrolysable tannins, also called ellagitannins, are the major chemical constituents of T. catappa leaves and that they have been linked to the antitumor (Chen et al., 2000) and antioxidant activities attributed to this plant (Kinoshita et al., 2007). The same group of compounds has been associated with a strong antimicrobial activity against bacteria and yeast (Lim et al., 2006). This effect was suggested to involve the precipitation of proteins or the removal of metal and hydrogen ions from microbial enzymes, thereby modifying vital metabolic processes in these microrganisms (Okuda, 2005; Costa et al., 2008). In addition to these hydrolysable tannins, both gallic acid and the glycosylated phenols found in FBuOH may contribute to its antifungal activity. This is supported by findings in that the gallic acid obtained from the air-dried mature Galla rhois methanolic extract and other plants is antimicrobial (Ahn et al., 2005; Chanwitheesuk et al., 2007). Glycosylated phenolic compounds isolated from natural sources possess antifungal properties of interest. Particularly, phenolic acids have shown promising in vitro and in vivo activity against Candida species (Ozcelik et al., 2011; Teodoro et al., 2015). Flavonoids, especially their glycosides, are of great general interest due to their diverse bioactivity. Flavonoid C-glycosides, obtained from different types of plans, have shown significant antioxidant activity, anticancer, and antitumor activity, hepatoprotective activity, anti-inflammatory activity, anti-diabetes activity, antiviral activity, antibacterial, and antifungal activity, and other biological effects (Xiao et al., 2016).

In conclusion, the FBuOH obtained from the TcHE of *T. catappa* L. leaves is a potent antifungal against *Candida* spp., rich in hydrolysable tannins, gallic acid and glycosylated phenols. Also, the low cytotoxic effects of TcHE support the use of FBuOH or its derived sub-fractions and/or compounds as alternative therapies for *Candida*-induced infections.

## Author contributions

CM conceived and designed the work and drafted the manuscript; AM, EF, and JS revising the work critically for important intellectual content and drafted the manuscript; AT was responsible for contact with patients and sample collection; AT and CM participated in all manipulations, and they analyzed and interpreted the results; ACRB and ES participated in plant collection and phytochemical tests; EM, AP, and PC helped with bio-guided fractionation and participated in all antifungal tests. LT and AM were responsible by chemical analysis and identification of components of FBuOH and its sub-fractions; AKDBF has given substantial contributions to the conception or design of the work and revised the manuscript. EF revised the language of manuscript. All authors read and approved the final manuscript.

### Conflict of interest statement

The authors declare that the research was conducted in the absence of any commercial or financial relationships that could be construed as a potential conflict of interest.

## Abbreviations

AIDS: Acquired Immunodeficiency Syndrome
ANOVA: Analysis of Variance
ATB: Automation Triple Resonance Broadband
ATCC: American Type Culture Collection
*BSTFA*+*TMCS*: N,O-bis (trimethylsilyl) trifluoroacetamide with trimethylchlorosilane
CFU: Colony Forming Units
CLSI: Clinical and Laboratory Standards Institute
DMSO: Dimethyl Sulfoxide
FAcOEt: Ethyl acetate fraction
FBuOH: n-butanol fraction
FHEX: Hexane fraction
GC/MS/EI: Gas Chromatography coupled to Mass Spectrometry withElectron Impact ionization
HIV: Human Immunodeficiency Virus
^1^HNMR: Nuclear Magnetic Resonance of Hydrogen
HPLC/UV: High-performance liquid chromatography coupled with Ultraviolet spectroscopy
HPLC-MS/MS/ES^+^: Liquid Chromatography coupled to Electrospray Ionization Mass Spectrometry in positive mode
MIC: Minimum Inhibitory Concentration
MTT: 3-(4, 5-dimethylthiazol-2-yl)-2,5-diphenyl tetrazolium bromide
NIST/EPA/NIH: National Institute of Standard and Technology Mass Spectral Library/Environmental Protection Agency/National institutes of Healthy
OPC: Oropharyngeal Candidiasis
PBMC: Human Peripheral Blood Cell
RPMI: Roswell Park Memorial Institute medium
SD: Standard Deviation
SDA: Sabouraud Dextrose Agar
SF: Sub-fractions
SF10-FbuOH: FBuOH sub-fraction 10
TcHE: *Terminalia catappa* Hydroalcoholic Extract
TIC: Total Ion Chromatogram
TMS: Tetramethylsilane
UFMA: Federal University of Maranhão.
